# Hand Matters: Left-Hand Gestures Enhance Metaphor Explanation

**DOI:** 10.1037/xlm0000337

**Published:** 2017-01-12

**Authors:** Paraskevi Argyriou, Christine Mohr, Sotaro Kita

**Affiliations:** 1 University of Birmingham; 2 University of Lausanne; 3 University of Warwick

**Keywords:** metaphor, gesture handedness, brain hemispheric lateralization, right hemisphere, mouth asymmetry

## Abstract

Research suggests that speech-accompanying gestures influence cognitive processes, but it is not clear whether the gestural benefit is specific to the gesturing hand. Two experiments tested the “(right/left) hand-specificity” hypothesis for self-oriented functions of gestures: gestures with a particular hand enhance cognitive processes involving the hemisphere contralateral to the gesturing hand. Specifically, we tested whether left-hand gestures enhance metaphor explanation, which involves right-hemispheric processing. In Experiment 1, right-handers explained metaphorical phrases (e.g., “to spill the beans,” beans represent pieces of information). Participants kept the one hand (right, left) still while they were allowed to *spontaneously* gesture (or not) with their other free hand (left, right). Metaphor explanations were better when participants chose to gesture when their left hand was free than when they did not. An analogous effect of gesturing was not found when their right hand was free. In Experiment 2, different right-handers performed the same metaphor explanation task but, unlike Experiment 1, they were *encouraged* to gesture with their left or right hand or to not gesture at all. Metaphor explanations were better when participants gestured with their left hand than when they did not gesture, but the right hand gesture condition did not significantly differ from the no-gesture condition. Furthermore, we measured participants’ mouth asymmetry during additional verbal tasks to determine individual differences in the degree of right-hemispheric involvement in speech production. The left-over-right-side mouth dominance, indicating stronger right-hemispheric involvement, positively correlated with the left-over-right-hand gestural benefit on metaphor explanation. These converging findings supported the “hand-specificity” hypothesis.

Imagine two people talking face-to-face. Now imagine a person talking on the phone. One thing is common: whether seen by others or not people often spontaneously produce hand gestures to accompany their speech. This shared feature between the two imagined settings illustrates the dual functions of gestures: (a) gestures express information valuable for the listener, and thus play an important role in how people communicate (Hostetter, 2011), and (b) gestures can influence cognitive processing of the speakers themselves and determine the contents of their thoughts and speech (de Ruiter, 1995; Kita, 2000; Rauscher, Krauss, & Chen, 1996). The current study focused on the latter, so-called self-oriented functions of gestures, and investigated whether they can be specific to the gesturing hand.

Literature on gestures suggests that speech and gesture often co-occur and coexpress the speakers’ message as a composite signal (Engle, 1998; Kelly, Ozyurek, & Maris, 2010; Kendon, 2004). Speech and gesture are tightly linked behaviors at various levels of language structure such as phonetics, syntax, semantics, and pragmatics (Iverson & Thelen, 1999; Kita & Ozyürek, 2003; McNeill, 1992). This close relationship between language and gesture has drawn scholars’ attention in a wide range of research topics such as the embodied nature of language processing (Glenberg & Kaschak, 2002; Hostetter & Alibali, 2008), the role of the body in understanding and representing abstract thought (Cienki & Müller, 2008; Lakoff & Johnson, 1980a; Mittelberg & Waugh, 2009), and the gestural origin hypothesis of language evolution (Arbib, 2005; Corballis, 2003). The current study investigated this relationship between language and gesture^1^ with a focus on the causal link “from-gesture-to-language,” and the aim to better characterize the role of the body in representing abstract thought and how gestures help linguistic expression of abstract knowledge.

Various theoretical accounts have been proposed to explain the gestural benefit in the gesturer’s mental processes (for a review see Kita, Chu, & Alibali, 2016): lexical retrieval (Krauss & Hadar, 2001; Pine, Bird, & Kirk, 2007; Rauscher et al., 1996), imagery maintenance (de Ruiter, 1995; Wesp, Hesse, Keutmann, & Wheaton, 2001), conceptualization for speaking (Alibali & Kita, 2010; Alibali, Kita, & Young, 2000; Hostetter, Alibali, & Kita, 2007; Kita, 2000; Melinger & Kita, 2007), and working memory (Goldin-Meadow, Nusbaum, Kelly, & Wagner, 2001). However, whether the right versus left hand has different facilitative effects remains to be explored. In particular, no studies have investigated whether gestural benefit is specific to the gesturing hand (left or right) for some linguistic tasks and relates to the hemispheric dominance for language processing.

It is plausible that a certain gestural benefit on language processing is specific to one hand for five reasons. First, language is a lateralized function of the brain (Broca, 1861; Wernicke, 1874), and second, the cortical control of hand movements is contralateral; that is, the right hemisphere mostly controls hand movements with the left hand and the left hemisphere mostly controls hand movements with the right hand (Cincotta & Ziemann, 2008). Third, spontaneous hand choice for gesturing is associated with which hemisphere is language dominant (Kimura, 1973a, 1973b). Right-handed healthy adults with strong left lateralization for language (measured with a right-ear advantage in a dichotomous listening task) produce more right-handed gestures than left-handed gestures in a free speech production task (Kimura, 1973a). Additionally, left-handed adults with a right-ear advantage produced more right-handed gestures compared to left-handed adults with a left-ear advantage (Kimura, 1973b). Fourth, evidence from language development also suggests that gesture and speech are developed hand-in-hand in the left hemisphere. For example, Mumford and Kita (2016) showed that 10- to 12-month-old infants who are more strongly right-handed when pointing have a larger vocabulary. Fifth, studies on action and gesture comprehension have also indicated that the left hemisphere is involved in processing the meaning of actions (Decety et al., 1997) or semantically integrating speech and gesture (Willems, Ozyürek, & Hagoort, 2007). Taken together, each hand has processing link to the contralateral hemisphere, which makes it likely that gesture facilitates language processing in a hand-specific way. Studies on split-brain patients suggest that the left hemisphere is not the only one responsible for gesture production. Kita and Lausberg (2008) showed that split-brain patients (with either left-hemisphere dominant or bilateral language representation) produced gestures with spatial content with both left and right hands. That is, even the nonlanguage-dominant right hemisphere could generate gestures independently from left-hemispheric speech production. Lausberg, Zaidel, Cruz, and Ptito (2007) found that split-brain patients preferentially used their left hand for beat gestures and shrugs. Other studies on split-brain patients provided converging results (Lausberg, Davis, & Rothenhaüsler, 2000; McNeill, 1992; McNeill & Pedelty, 1995). As beats are thought to be linked to speech prosody (Krahmer & Swerts, 2007), this finding indicates that the right hemisphere dominance in prosody production (Lindell, 2006) led to the left-hand preference for this type of gestures.

Semantic processing may be a fruitful area when investigating the hand-specificity of gestures’ self-oriented functions because semantics partially determines hand choice for gesture production. For example, Lausberg and Kita (2003) showed that spatial aspects of a message determined the choice of the right or left hand for gesturing (e.g., use of left hand to gesturally depict an object moving in the relative left position). In addition, Casasanto and Jasmin (2010) found that speakers used their dominant hand (either left or right) to represent messages with positive connotations in political debates. This finding suggested that emotional valence (positive–negative), and the way right- and left-handers represent valence (e.g., the dominant side, either left or right, is positive) may determine hand choice for gesturing (Casasanto & Jasmin, 2010).

To summarize, gesture production can influence the gesturer’s cognitive processes; that is, gesture has self-oriented functions. Spontaneous hand choice for gesturing is associated with hemispheric dominance for language processing and with types of meanings and functions of gestures. However, it is not clear whether gestures’ self-oriented functions can be specific to the right hand or the left hand. In order to investigate this question, the current study focused on semantic processing that crucially involves the right hemisphere, namely, metaphor. We focused on metaphor processing because (a) it crucially involves the right hemisphere (Jung-Beeman, 2005), and (b) it causes increased preference of left- compared to right-hand gesturing (Kita, de Condappa, & Mohr, 2007).

Different types of evidence (e.g., patient, neuroimaging, behavioral studies) support the idea that the right hemisphere is particularly involved for metaphor processing. Studies of patients with right and left hemisphere lesions performing metaphor tasks (Brownell, Simpson, Bihrle, Potter, & Gardner, 1990; Winner & Gardner, 1977) suggested that the left hemisphere is not adequate for the processing of every linguistic meaning, such as metaphorical meaning. Additionally, neurophysiological evidence from positron emission tomography scan studies (Bottini et al., 1994) and functional MRI studies (Mashal, Faust, & Hendler, 2005; Mashal, Faust, Hendler, & Jung-Beeman, 2007) of healthy adults processing metaphorical phrases showed a shared activation of a core bilateral network for metaphorical and nonmetaphorical phrases, and a special role of the right hemisphere for the metaphorical ones. Finally, divided visual field studies using metaphorical relationships at word and sentence levels showed a right hemisphere advantage. Anaki, Faust, and Kravetz (1998) used semantic priming for word pairs related literally (e.g., “stinging”–“mosquito”) and metaphorically (e.g., “stinging”–“insult”). Metaphorically related targets showed faster processing when presented in the left visual field (right hemisphere) than the right visual field, and the pattern was reversed for the literal targets. Similarly, Schmidt, DeBuse, and Seger (2007) found faster semantic judgment for metaphorical sentence endings (e.g., “the camel is a dessert”–“taxi”) when presented in the left than the right visual field, and the reversed pattern was found for literal sentence endings (e.g., “the camel is a dessert”–“animal”). Although some studies failed to provide such evidence (Rapp, Leube, Erb, Grodd, & Kircher, 2004, 2007), there is substantial support for the right-hemisphere hypothesis for metaphor (see Schmidt, Kranjec, Cardillo, & Chatterjee, 2010, for a review on the neural correlates of metaphor).

Metaphor processing in the right hemisphere triggers left-hand gesturing. In Kita et al. (2007) participants explained metaphorical phrases such as “to spill the beans,” and in the control conditions, they explained the meaning of concrete and abstract phrases with similar meanings (i.e., “to spill the marbles,” “to reveal something confidential”). They produced gestures spontaneously (the instruction did not mention gesture) during explanations and the proportion of left-hand gestures out of all unimanual gestures was higher in the metaphor condition than the concrete and the abstract condition. These results support the idea that language processes in the right hemisphere increase left-hand choice for gesturing. It is not clear, however, whether gestures with the left hand specifically enhance metaphor processing in the contralateral right hemisphere.

## Present Study

The present study tested whether gestures facilitate linguistic tasks, such as metaphor explanation, in a “hand-specific” manner due to the mutual influence between language hemispheric dominance and hand choice for gesturing. More specifically, we examined whether left hand gesturing improves performance in metaphor explanation tasks, and if so, whether this benefit relates to relative hemispheric involvement for linguistic tasks.

In Experiment 1, we tested whether spontaneous gesturing with the left hand is associated with improved performance in a metaphor explanation task. We manipulated gesture production by asking participants to perform the metaphor explanation task (same task as in Kita et al., 2007) while one hand is prohibited from movements and the other hand is free to gesture. Participants were asked to explain the metaphorical mapping underlying English phrases, such as “to spill the beans” (meaning “to reveal a secret”): “Beans” represent secrets and “spilling” represents dispersion of information. Tasks using these phrases have been previously shown to engage metaphorical thinking and are thus likely to involve the right hemisphere (Argyriou, Byfield, & Kita, 2015; Kita et al., 2007). The explanations were rated for the level of metaphoricity, namely, how well participants described metaphorical mappings. This coding captures the key elements of metaphor processing, because metaphorical mappings are key parts of metaphor interpretation processes (Lakoff & Johnson, 1980a; Nayak & Gibbs, 1990). If hand matters and gestures support metaphor explanation in a “hand-specific” manner, then metaphor explanations should be of higher quality when participants spontaneously gestured with the left hand compared to not gesturing with it, while right-hand gesture presence/absence should make no difference.

Experiment 2 investigated whether left-hand gestures improve metaphor explanation by more directly manipulating the hand to produce gestures. Participants completed the same metaphor explanation task as in Experiment 1, but we explicitly encouraged them to gesture with their left hand only or right hand only or to not gesture at all. If gestures improve metaphor explanation in a “hand-specific” manner, then metaphor explanations should be of higher quality and metaphorical mappings should be explained more elaborately when participants were encouraged to gesture with their left hand compared to not gesturing.

Experiment 2 also aimed to link the left-hand specific gestural benefit on metaphor processing with processing in the contralateral hemisphere by an individual difference approach. In order to do so, we measured mouth asymmetry during speaking from each participant as an indicator of which hemisphere is dominant in speech production.

Mouth asymmetry is one of the behavioral measures for relative hemispheric involvement during different cognitive tasks. For example, Graves and Landis (1985, 1990) showed that the right side of the mouth opened wider than the left during propositional speech (e.g., spontaneous speech, word list generation), reflecting the left hemisphere cerebral involvement for speech production. In contrast, during automatic speech (e.g., singing, counting) or emotional expressions (e.g., spontaneous smiles; Wyler, Graves, & Landis, 1987), which are both thought to particularly involve the right hemisphere (see for a review Lindell, 2006), the left side of the mouth opened wider than the right. In addition, Argyriou et al. (2015) showed that the right-side dominance in mouth opening was reduced for males during explanation of metaphorical phrases as compared to nonmetaphorical phrases (same tasks as in the present study), and this reduction was larger for content words that carry meaning (e.g., nouns, verbs) than for function words (e.g., conjunctions, determiners). This suggested that mouth opening asymmetry is sensitive to hemispheric differences in semantic processing involved in metaphor explanation.

We collected mouth asymmetry measurements from the participants in Experiment 2 during speech production in a separate explanation task. We predicted that the left-hand gestural benefit on metaphor explanation should be stronger for those who show a stronger right hemisphere involvement in speech production during explanation tasks. When one hemisphere (e.g., right) is strongly involved in verbal explanations, gestures with the contralateral hand (e.g., left-hand gestures rather than right-hand gestures) should facilitate verbal explanations.

## Experiment 1

### Method

#### Participants

Thirty-two right-handed, male, native English speakers (monolinguals at least until the age of 5 years; age at testing *M* = 22.35 and *SD* = 4.82) participated in the experiment for course credit. Handedness was assessed with a 12-item questionnaire based on the Edinburgh Handedness Inventory (Oldfield, 1971). Two bimanual items (from Oldfield’s long list) were added to his recommended 10-item questionnaire to equate the number of unimanual and bimanual items (see Text S1 in the supplementary material for the questionnaire). Each “left” answer was scored with 0, each “either” answer with 0.5, and each “right” answer with 1. A total score of 8.5 and above determined right-handedness (*M* = 11.12 and *SD* = 1.16). All of them were recruited and tested at the University of Bristol. We focused on male speakers because they exhibit bilateral representation of language processing less frequently than women (McGlone, 1980), while language processing, can be modulated by hormones, and hence be less stable in women (Hausmann & Güntürkün, 2000).

#### Stimuli

We used 12 English phrases with metaphorical meaning identical to the ones used in the metaphorical condition in Kita et al. (2007; see Appendix A).

#### Procedure

Participants were tested individually. They were seated on a chair, which was located between two tables of the same height (71 cm tall). The experimenter was facing the participant, and the video camera (Sanyo high-definition camera, Sanyo Xacti VPC-HD1000, Japan) was placed next to the experimenter. Stimuli were presented one by one on a white sheet of paper (72-point Times New Roman font), which was held by the experimenter until the participant started the description.

**Figure 1 fig1:**
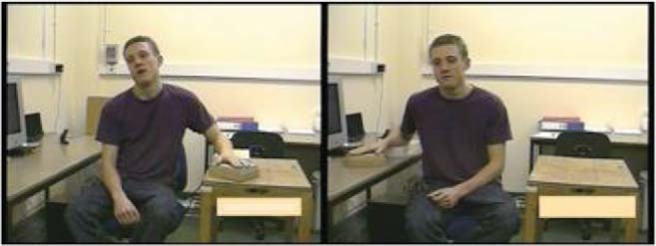
Experimental conditions in Experiment 1: Right hand free (left panel), left hand free (right panel). See the online article for the color version of this figure.

Participants were instructed to explain the meaning of the 12 metaphorical phrases (see Appendix A) as if they were explaining it to a nonnative English speaker (the task was the same as in the metaphorical condition in Argyriou et al., 2015 and Kita et al., 2007). The hand that is free to gesture was manipulated within participant. In order to immobilize the one hand, participants were asked to place the right or left hand on a device measuring skin conductance. They were given no instruction about gesturing with their free hand. Therefore, they *spontaneously* produced gestures with their free hand in some trials but not in others (see Figure 1). Participants were debriefed about the purpose of the hand’s immobilization after the experiment and permission to use the data was given.[Fig-anchor fig1]

There were two practice trials preceding the main trials. In the main trials, the hand free to gesture was manipulated within participant, and each participant completed a block of six trials for the right-hand-free condition and another block of six trials for the left-hand-free condition. The order of which hand was free to gesture first was counterbalanced across participants (i.e., half the participants explained six phrases while they were free to gesture with their left hand, and then they explained six phrases while they were free to gesture with their right hand; for the other half of the participants, the condition order was reversed). The 12 stimuli were presented in one of the two fixed orders: The order of the stimuli (forward–reverse) was counterbalanced across participants.

#### Coding

The verbal responses from the task were transcribed and coded for level of metaphoricity. The level of metaphoricity was measured based on whether the explanations included an explicit link between the literal and metaphorical meanings, and whether participants explicitly referred to the mapping and correspondences between the source and target domains of the conceptual metaphor underlying each phrase (following the conceptual metaphor theory; Lakoff & Johnson, 1980a; Nayak & Gibbs, 1990). The stimulus phrases were idiomatic, which may not always activate the right hemisphere. For example, Papagno, Oliveri, and Romero (2002) used repetitive transcranial stimulation while participants matched the meaning of an idiom to a picture. They found no evidence that right temporal lobe stimulation affected response times and accuracy. However, the measurement in the current study captures how well participants actively analyzed the literal and metaphorical meaning, and they established a metaphorical mapping between distant semantic relations. Such a process is considered crucial for the right-hemispheric involvement for metaphorical processing (Jung-Beeman, 2005). More specifically, a 0 rating indicated that the explanation did not contain words or phrases referring to the source domain of the relevant conceptual metaphor; therefore, there was no metaphorical cross-domain mapping; a rating of 1 indicated that the explanation contained words or phrases that might be construed as references to the source domain, but the references were ambiguous, and the mapping between the two domains implicit; a rating of 2 indicated that the explanation contained words or phrases that clearly referred to the source and target domains, and the mapping was explicit. Each code (0, 1, 2) was attributed to the entire verbal response^2^ (i.e., one code per trial). Text S2 in the supplementary material presents the detailed coding manual.

Video recordings from the two gesturing conditions were analyzed using ELAN software (developed by the Max Planck Institute for Psycholinguists in Nijmegen, the Netherlands). Each trial was classified into two types: spontaneous gesture present versus absent. For the purposes of the current study, we did not include self-adaptors and beat gestures, because they do not represent semantic information related to speech (Lavergne & Kimura, 1987). That is, trials including at least one representational or conduit or palm-revealing gesture were coded as “spontaneous gesture present.”

#### Reliability of coding

Two coders, “blind” to the research hypothesis and experimental conditions, were trained and independently coded all the verbal responses in terms of metaphoricity. Coding of metaphoricity matched between the two coders 87% of the time (Cohen’s weighted kappa, κ_w_, = .791, *p* < .001). The coders discussed their disagreements and agreed on one coding, which was used for the final analysis reported here.

#### Design

The dependent variable was the level of metaphoricity in participants’ explanations. The experiment had a 2 × 2 factorial design with two independent variables (within-subjects design): hand free (left, right) and presence/absence of spontaneous gesture.

#### Notes on mixed-effect models

We used linear mixed-effects (LME) models with subject and item as random factors, and the packages *lme4* and *multcomp* in the R Project for Statistical Computing environment, Version 3.1.1 (Bates & Sarkar, 2012; Hothorn, Bretz, & Westfall, 2012; R Development Core Team, 2011). All mixed effects regressions were carried out with “lmer()” function specifying that maximum likelihood (rather than restricted maximum likelihood) is used (needed to get a more valid likelihood ratio test of the full against the null model). Random effects structure was kept maximal as long as model convergence was reached (for a discussion about random effects structure and simplification, see Barr, Levy, Scheepers, & Tily, 2013). We obtained *p* values for fixed effects following the likelihood ratio test approach for model comparison and we always reported the maximal model following a design-driven approach for confirmatory analyses. Tests of further contrasts of our interests were carried out based on a priori predictions using the generalized linear hypothesis test with correction for multiple comparisons of means (Tukey contrasts) using the “glht()” function. The R code for all the models and comparisons reported can be found in Text S3 in the supplementary material.

### Results

Out of the 384 trials in total in the task, 8% were excluded as failed trials, that is, when the participants did not follow the instruction (i.e., they moved the prohibited hand; in four trials, they moved the right hand; in three trials, the left) or when they did not know the phrases (23 trials).

**Figure 2 fig2:**
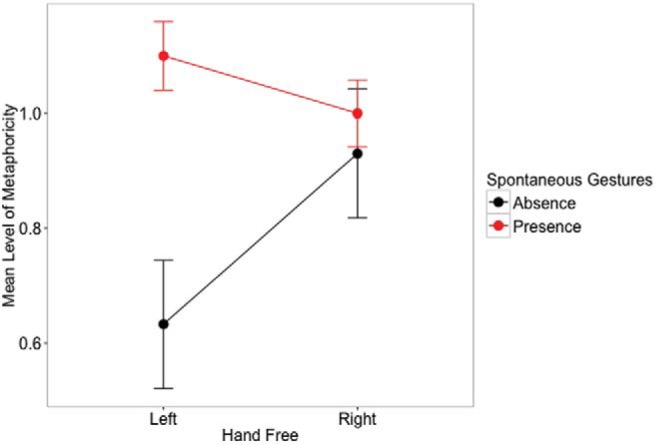
Mean levels of metaphoricity in speech in the four gesturing conditions (Experiment 1). Error bars represent 1 *SEM*. See the online article for the color version of this figure.

We ran linear mixed effect models following the specifications in the section Notes on Mixed-Effect Models. We fitted LME model to the measurement of the level of the metaphoricity (see Figure 2 for the means). The model included two fixed-effect factors and the interaction between the two. The one fixed factor was the hand free (left, right; dummy coded; “right” was the reference category). The second fixed factor was presence/absence of spontaneous gestures (dummy coded; “absence” was the reference category). We included random intercepts and slopes by subjects and items (phrases) for the main effects and interaction of the fixed-effect factors.[Fig-anchor fig2]

Model estimates are reported in Table 1. We compared the maximal model with the reduced model including the main effects only (same random effect structure). Adding the interaction significantly improved the model fit: χ^2^(1) = 5.158, *p* = .023 (see Figure 2). Simultaneous tests for general linear hypotheses (Tukey contrasts) revealed that the contrast between presence and absence of spontaneous gestures was significant for the left hand, but not for the right hand (see Table 2). Thus, spontaneously gesturing with the left hand is associated with a higher level of metaphoricity in metaphor explanation compared to not gesturing with it by choice.[Table-anchor tbl1][Table-anchor tbl2]

### Discussion

We examined whether spontaneous gesturing by a specific hand is associated with improved performance in a metaphor explanation task. In the left-hand-free condition, metaphoricity was higher for trials with spontaneous gesturing than those without. However, in the right-hand-free condition, such a relationship between performance and gesturing was not found. This result points to the “(right/left) hand-specificity” hypothesis for gestures’ self-oriented functions: The benefit of producing gestures is specific to one hand for some tasks. The result also suggests that the specific hand for which gesturing is beneficial is linked to cognitive processes involving the contralateral brain hemisphere. The left-hand specificity observed in the metaphor explanation task is compatible with the idea that the right hemisphere plays a crucial role in metaphor processing (Anaki et al., 1998; Jung-Beeman, 2005).

Experiment 1 is, however, limited in two ways. First, in Experiment 1, participants were free to spontaneously gesture or not. Therefore, we cannot distinguish whether gesture led to a higher level of metaphoricity or better metaphor explanations led to gesture. To address this issue, in Experiment 2, we manipulated presence versus absence of gesturing for each hand to see if this impacts performance. Second, Experiment 1 did not provide any data related to hemispheric involvement for language processing. To address this issue, in Experiment 2, we took an individual difference approach, in which we took a behavioral measurement (other than gesturing) indicative of relative hemispheric involvement for language processing to see if this measurement correlates with the degree to which gestural benefit is specific to one hand.

## Experiment 2

This experiment had two goals. First, we examined whether producing left-hand gestures improves the performance of metaphor explanation. Participants were asked not to move one hand (right or left), but, unlike Experiment 1, they were encouraged to produce gestures with the free hand. They also performed the metaphor explanation task while instructed not to gesture with either hand. Second, we examined whether the degree to which the gestural benefit is specific to the left hand is correlated with an index of relative contributions of the two hemispheres for speech production. To obtain this additional index, participants completed a separate explanation task (while gestures were prohibited), in which we video recorded their mouth movements during speaking. We measured which side of the mouth opens more widely as an indirect measurement of the relative strength of the two hemispheres’ involvement in speech production (Graves & Landis, 1985, 1990).

We predicted that, parallel to the finding in Experiment 1, participants would give better metaphor explanations when they gestured with their left hand than when they did not gesture. We also predicted that relative left-hand gestural benefit would positively correlate with the relative right-hemispheric involvement during speech production as measured via the mouth asymmetry technique.

### Method

#### Participants

Thirty-one right-handed, male, native English speakers (monolinguals at least until the age of 5 years; age at testing: *M* = 20.35 and *SD* = 2.86) participated in the experiment for course credit or £4. They did not participate in Experiment 1. Handedness was assessed as in Experiment 1 (*M* = 10.9 and *SD* = 1.08; see Text S1 in the supplementary material for the questionnaire). None of the participants had any previous serious injury to the face or jaw. All of them were recruited and tested at the University of Birmingham.

#### Stimuli

For the main metaphor explanation gesture elicitation task, we used 18 English phrases with metaphorical meaning. We added six phrases to the list of metaphorical stimuli used in Experiment 1, because we added an experimental condition (the no-hand-free condition) and we wanted to keep the number of items per condition (six items) the same as in Experiment 1. For the mouth asymmetry task, we created three (plus one reserve item in case one phrase was unknown) additional metaphorical and concrete phrases (see Appendix B).

#### Procedure

**Figure 3 fig3:**
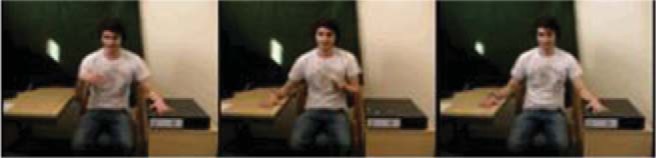
Experimental conditions in Experiment 2: Right hand gesturing (left panel), left hand gesturing (middle panel), no gesturing (right panel). See the online article for the color version of this figure.

The procedure was essentially the same as in Experiment 1 with few alterations. Participants were instructed to explain the meaning of the 18 metaphorical phrases (see Appendix B) as if they were explaining it to a nonnative English speaker. The hand used for gesturing was manipulated within participant. For the right-hand gesturing condition and the left-hand gesturing condition, participants were told to place one of their hands on the indicated marks (white sticky dots) on the surface of the table(s), and to keep it still for the whole procedure. For the no-hand-free condition (the total prohibition condition), participants were asked to place both hands on the table (see Figure 3). The no-hand-free condition was necessary to compare metaphoricity while gesturing versus not gesturing. For the gesturing conditions, participants received gesture encouragement instructions (i.e., the experimenter asked them, “Please use your free hand to gesture while speaking”). Gesture encouragement has been used in a number of recent studies (Broaders, Cook, Mitchell, & Goldin-Meadow, 2007; Chu & Kita, 2011; Cook, Yip, & Goldin-Meadow, 2012). It allowed us to directly test the “from-gesture-to-metaphor” casual direction and to include most of the trials in the analysis as gesturing trials. Participants were debriefed about the purpose of the hands immobilization after the experiment and permission to use the data was given.[Fig-anchor fig3]

There were two practice trials. In the main trials, the hand for gesturing was manipulated within participant, and each participant completed a block of six trials for each of the three conditions (right hand gesturing, left hand gesturing, no hand gesturing). The order of the conditions was counterbalanced across participants. The 18 stimuli were presented in one of the two fixed orders: the order of the stimuli (forward–reverse) was counterbalanced across participants.

The mouth asymmetry task followed the metaphor explanation gesture elicitation task. In the mouth asymmetry task, participants were instructed to explain the three metaphorical phrases (see Appendix B; i.e., explain the mapping of the literal meaning to the metaphorical meaning), just as in the main metaphor explanation gesture elicitation task. They also explained the meaning of three concrete phrases (see Appendix B) and were instructed to be as elaborate as possible. During the explanations use of both hands were prohibited. Hand prohibition was necessary in order to collect a pure measurement of participants’ hemispheric involvement for speech production without any influence from hand movement. The order of the tasks (concrete–metaphorical) was counterbalanced across participants. Video-recording zoomed-in on the face area.

#### Coding

The verbal responses from the main metaphor explanation and gesture elicitation task were transcribed and coded for level of metaphoricity exactly in the same way as in Experiment 1 (see Text S2 in the supplementary material for the detailed coding manual).

Video recordings from the two gesturing conditions in the main task were analyzed using ELAN software (developed by the Max Planck Institute for Psycholinguists, Nijmegen, the Netherlands). They were coded on a trial-by-trial basis to locate the existence of at least one gesture type, using the coding scheme by Chu, Meyer, Foulkes, and Kita (2014), that is, representational gestures (e.g., hand movements depicting shape, motion and action or deictically indicate location), palm-revealing gestures (e.g., palm rotates to show uncertainty or that speaker has nothing to say or), conduit gestures (e.g., hand moves toward listener as if speaker is conveying a clear message), and other (e.g., small biphasic movements/beats). See the supplementary material in Chu et al. (2014) for more detail.

**Figure 4 fig4:**
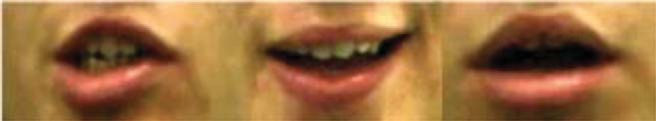
Examples of maximum mouth opening asymmetry in Experiment 2. Right-sided asymmetry (left panel), left-sided asymmetry (middle panel), both sides equally open (right panel; “left-sided” and “right-sided” refer to participants’ left and right). See the online article for the color version of this figure.

Video recordings from the mouth asymmetry task were analyzed on a frame-by-frame basis using ELAN software to identify the maximum mouth openings in each phrase explanation. One maximum opening was defined as the widest point the mouth opens, from when the lips open to when the lips rested or when the lips met completely. We coded the laterality at each maximum mouth opening. The options for laterality classification were right-side dominant (the right side of the mouth opens wider than the left), left-side dominant (the left side of the mouth opens wider than the right), or sides equally open (see Figure 4 for examples). Maximum openings for filled-pauses were coded, but not the ones for nonspeaking purposes (e.g., smile) or the ones while participants were repeating the phrase to be explained. We coded the first 30 mouth openings per condition (metaphorical–concrete) per participant (the first 10 mouth openings from each explanation; following Graves, Goodglass, & Landis, 1982 who also coded the first 10 successive lip openings with word production). In total, we coded 930 mouth openings in the metaphorical task and 915 in the concrete task (four participants gave short explanations in the concrete task, and, thus, we could only obtain less than 30 mouth openings per condition). Text S4 in the supplementary material presents the detailed coding manual.[Fig-anchor fig4]

#### Reliability of coding

Two coders, “blind” to the research hypothesis and experimental conditions, were trained and independently coded all the verbal responses in terms of metaphoricity. Coding of metaphoricity matched between the two coders 92% of the time (Cohen’s weighted kappa, κ_w_ = .902, *p* < .001). The coders discussed their disagreements and agreed on one coding, which was used for the final analysis reported here.

The first author coded the video recordings from the two gesturing conditions in terms of the existence (or absence) of at least one gesture type. An additional coder, “blind” to the research hypothesis and experimental conditions, was trained and independently coded 24% of the video recordings. All answers from seven randomly selected participants were coded (in total 84 trials were double coded). Coding matched between the two coders 98% of the time for the coding of trials with at least one representational gesture; 85% of the time for palm-revealing gesture; 96% of the time for conduit gesture; 81% of the time for other gesture. Note that measurement of agreement (kappa statistic) was not calculated because the random selection of cases for second coding led to a constant value (either absence or existence of particular gesture type for all 84 trials) for a variable upon which kappa is calculated. The first coder’s original coding was used for the descriptive statistics reported.

The first author coded the video recordings from the mouth asymmetry task in terms of laterality of mouth openings. An additional coder, “blind” to the research hypothesis and experimental conditions, was trained and independently coded 22% of the data in terms of right, left or equal dominance of mouth openings. All mouth openings from seven randomly selected participants were coded (in total 414 maximum mouth openings were double coded). Coding of mouth opening dominance matched between the two coders 91% of the time (Cohen’s κ = .854, *p* < .001). The first coder’s original coding was used for the analysis reported.

#### Design and measurements

The dependent variable from the main metaphor explanation gesture elicitation task was the level of metaphoricity in participants’ explanations. The independent variable (within-subjects design) “hand free” had three levels (left, right, no hand). Comparisons across these conditions would confirm gestures’ facilitative role on metaphor explanation and assess our “hand-specificity hypothesis” for this benefit.

Next, we measured the relative hemispheric involvement for speech production via the mouth asymmetry technique, while participants explained concrete and metaphorical phrases in a separate task. We computed a left-sided dominance in mouth openings using the following formula: (L – R)/(L + R + E), where L, R, and E are the numbers of left-side-dominant, right-side dominant, and equal mouth openings, respectively (Argyriou et al., 2015; Holowka & Petitto, 2002). Thus, a positive mean score indicated more instances of left-side dominant mouth openings (relative right-hemispheric involvement) and a negative mean score indicated more instances of right-side dominant mouth openings (relative left-hemispheric involvement).

Finally, we calculated a left-over-right-hand gesturing advantage index from the main metaphor explanation gesture elicitation task: the average level of metaphoricity when gesturing with the left hand minus the average level of metaphoricity when gesturing with the right hand. Thus, a high and positive mean score indicated that participants were more metaphoric when gesturing with their left hand compared to the right (left-over-right-hand gesturing advantage on metaphoricity). We argue that this difference score is a better measurement for the correlational analysis, compared to the metaphoricity scores in one of the gesturing conditions or other difference scores (e.g., a left-over-no-hand gesturing advantage on metaphoricity) for the following reasons. The gestural benefit on metaphor explanation could be assessed in absolute terms, that is, only for one hand (e.g., how metaphoric subjects were when gesturing with the left hand). However, the mouth asymmetry score is about *relative* dominance of the two sides (e.g., the left or the right side opens wider). Thus, mouth asymmetry cannot be assessed only on one side. Consequently, the measurement of gestural benefit to be correlated with the mouth asymmetry score should also be about relative dominance of the two sides (i.e., hands), namely, the difference score (left-over-right hand gesturing advantage on metaphor explanation).

### Results

Out of the 558 trials in the main task, 4% were excluded as failed trials; that is, when the participants did not follow the instruction (i.e., no gesture production when encouraged to gesture with the right or left hand) or when they did not know the phrases.

Out of the 354 gesturing trials, 99% included at least one representational gesture, 23% included at least one palm-revealing gesture, 7% included at least one conduit gesture; 18% included at least one “other” gesture—comprising mainly beat and metacognitive gestures. Thus, the instruction to produce gestures was effective and gestures were predominantly representational gestures.

**Figure 5 fig5:**
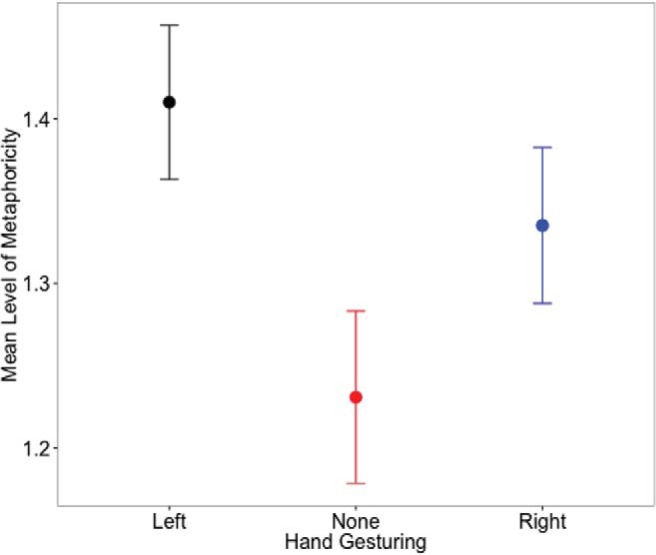
Mean levels of metaphoricity in speech in the three gesturing hand conditions (Experiment 2). Error bars represent 1 standard error of the means. See the online article for the color version of this figure.

We fitted LME model to the measurement of the level of the metaphoricity in the same way as in Experiment 1 (see Figure 5 for the means). The model included one fixed-effect factor: hand free (left, right, no hand; “no hand” was the reference category). We included random intercepts and slopes by subjects and items (phrases) for the fixed-effect factor. The R code for all the models and comparisons reported can be found in Text S5 in the supplementary material.[Fig-anchor fig5]

Model estimates are reported in Table 3. We compared the model with the null model with no fixed-effect factors (same random effect structure). Adding the effect of hand free for gesturing (left, right, none) improved the model fit: χ^2^(2) = 8.355, *p* = .015 (see Figure 5). Simultaneous tests for general linear hypotheses (Tukey contrasts; see Table 4) revealed that gestures with the left hand increased the level of metaphoricity in metaphor explanations as compared to not gesturing at all. [Table-anchor tbl3][Table-anchor tbl4]

**Figure 6 fig6:**
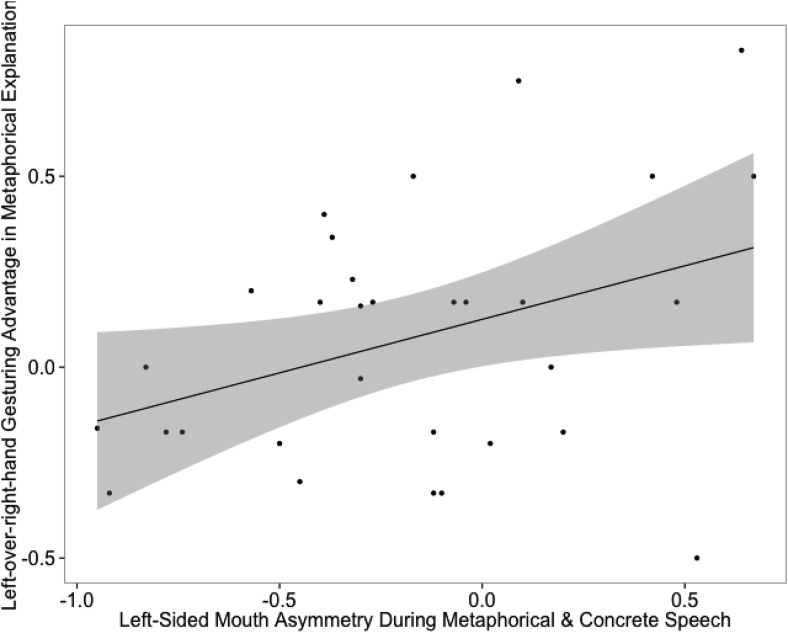
Scatterplot for the positive correlation between the averaged index of left-sided mouth asymmetry during speech and the left-hand gesturing advantage in metaphorical explanation (Experiment 2). The gray area represents 95% confidence limits.

Next, we investigated how mouth asymmetry during speaking (as described in the Design and Measurements section) related to the left-over-right-hand gesturing advantage. Though the left-side dominance in mouth opening was stronger for metaphorical phrases than concrete phrases (see Text S6 in the supplementary material), the degrees of the left-side dominance in the two types of phrases were highly correlated, *r*(29) = .829, *p* < .001, 95% confidence interval (CI) [.672, .914]. Thus, we used the average of the left-side dominance scores in the two types of phrases as a general indicator of right-hemispheric involvement in speech production (due to the high correlation, using the left-side dominance score from the metaphorical or the concrete phrases only yielded the same results). Crucially, the averaged left-side dominance in mouth openings for speech production (range = −.95 to .67) positively correlated with the left-over-right-hand gesturing advantage in metaphoricity (range = −.50 to .83), *r*(29) = .377, *p* = .036, 95% CI [.027, .645] (see Figure 6). Thus, the participants who had a stronger right-hemispheric involvement for speech production tended to have a larger left-over-right-hand gesturing advantage in metaphor explanations.[Fig-anchor fig6]

### Discussion

There were two key findings. First, gesturing with the left hand increased the level of metaphoricity in explanations compared to not gesturing at all (while we found no such evidence for the right hand). This confirms the causal direction “from-gesture-to-metaphor” that could not be concluded with certainty in Experiment 1. In addition, this result is compatible with the idea that gestures improve performance in tasks involving the hemisphere contralateral to the gesturing hand as metaphor processing crucially involves the right hemisphere (Anaki et al., 1998; Jung-Beeman, 2005).

Second, the relative left-over-right hand gesturing advantage for metaphor explanations was higher for those people who also had a stronger left-over-right side dominance in mouth opening during speaking, indicating relatively strong right hemisphere involvement in speech production (Graves & Landis, 1985, 1990). This latter finding provides evidence that gesturing with one hand is associated with improved performance in tasks involving processing in the contralateral hemisphere.

## General Discussion

The present study provided evidence for the “(right/left) hand-specificity” hypothesis for gestures’ self-oriented functions and for the idea that the benefit of gesturing with a particular hand relates with language lateralization for speaking.

The “hand-specificity” hypothesis was supported by the converging results of the two experiments. People produced better metaphor explanations when they produced gestures with the left hand by choice (Experiment 1) or by instruction (Experiment 2), as compared to when they did not. By contrast, we did not find such beneficial effect for gestures with the right hand. These results indicate that left-hand gestures facilitated metaphor processing. In Experiment 2, one may argue that prohibiting the left-hand movement (in the no-gesture condition) was detrimental to metaphor processing rather than gesturing with the left hand was beneficial (e.g., because remembering not to move the hand may have been distracting). However, this alternative explanation cannot explain the result of Experiment 1 because no-gesturing for the free hand in Experiment 1 was by choice not by prohibition. Taken together, we conclude that gesture facilitated metaphor processing in a manner specific to the gesturing hand; that is, in some tasks, either right or left hand serves self-oriented functions of gesture.

The idea that gestures with a specific hand facilitate processing in the contralateral hemisphere was supported by two findings, albeit the evidence is indirect. First, in both experiments, gesturing with the left hand (and not the right hand) facilitated metaphor explanation, which particularly involves the right hemisphere (Anaki et al., 1998; Jung-Beeman, 2005). Second, the left-side mouth dominance during speaking positively correlated with the left-over-right-hand gesturing advantage on metaphor explanation. This means that when the right-hemisphere is more strongly involved in speech production, left hand gestures can more readily support processes in the right hemisphere, such as metaphor explanation. Although the mouth asymmetry index is an indirect measurement (e.g., a direct physiological method could be used in the future to measure hemispheric involvement during speech production), it is an effective way to capture relative hemispheric involvement for different cognitive tasks (Argyriou et al., 2015; Graves et al., 1982; Graves & Landis, 1990).

This study goes beyond the previous literature in an important way. Several studies manipulated gesturing in order to assess gestures’ effect on speaking (Alibali & Kita, 2010; Goldin-Meadow et al., 2001; Rauscher et al., 1996). However, they did not investigate differential effects of right- versus left-hand gestures. Several studies showed that cognitive processes in a particular hemisphere are associated with gesturing with the contralateral hand (Kimura, 1973a, 1973b; Kita et al., 2007; Mumford & Kita, 2016). However, these studies did not investigate gestures’ causal role. Thus, the present study demonstrated, for the first time, that self-oriented functions of gestures can be specific to the gesturing hand (right/left) for some tasks. Furthermore, the present results also suggest that gesturing with a particular hand benefits the performance in linguistic tasks involving the hemisphere contralateral to the gesturing hand.

How, exactly, does this “(right/left) hand-specificity” hypothesis for gestures’ self-oriented functions work? We can speculate how in light of the conceptual metaphor theory (Lakoff & Johnson, 1980a, 1980b) and the fine–coarse semantic coding model (Beeman & Chiarello, 1998; Jung-Beeman, 2005) combined. Metaphor requires speakers to map two semantically distant concepts: a concrete concept from the source domain on to a more abstract one in the target domain (Lakoff & Johnson, 1980a). In the phrase “to spill the beans,” participants had to represent the abstract concept of *ideas* (target) in terms of the distant concrete concept of *objects* (source). The right hemisphere is more interconnected than the left hemisphere (i.e., the right hemisphere has more white matter and neuron connections than the left hemisphere; Jung-Beeman, 2005). For this reason, the right hemisphere is thought to be crucially involved in processing of coarse-grained semantic information and thus more distant semantic relationships (Jung-Beeman, 2005), such as metaphorical mappings. Producing gestures activates spatio-motoric information (Alibali & Kita, 2010; Alibali, Spencer, Knox, & Kita, 2011; de Ruiter, 1995; Hostetter & Alibali, 2008; Kita et al., 2016; So, Ching, Lim, Cheng, & Ip, 2014; Wesp et al., 2001). Producing left hand gestures should do so more strongly in the right hemisphere because the hand movements are predominantly controlled by the contralateral hemisphere (Cincotta & Ziemann, 2008). Therefore, left hand gestures should help “visualizing” the source domain representation, which makes it easier to discern the distant semantic relationship to the target domain of the metaphor, and allow speakers to represent the metaphorical mapping in speech more easily. That is, gestures with a particular hand can modulate the content of speech when the linguistic task particularly involves the hemisphere contralateral to the gesturing hand.

The present findings are also in line with the Information Packaging Hypothesis for self-oriented functions of gestures (Alibali et al., 2000; Hostetter et al., 2007; Kita, 2000; Melinger & Kita, 2007) and the gesture for conceptualization hypothesis (Kita et al., 2016), which state that gestures can help conceptual planning of the speech by activating spatiomotoric representations. We showed that left hand gestures help the conceptual mapping from the source domain to the target domain of metaphor, thereby influencing the course of thinking (Alibali et al., 2011) and the content of verbal output (Alibali & Kita, 2010; Rime, Schiaratura, Hupet, & Ghysselinckx, 1984).

## Conclusions

The present study has, for the first time, provided evidence for the “(right/left) hand-specificity” hypothesis for gestures’ self-oriented functions. Left-hand gestures (by choice and by instruction) enhanced metaphor explanations compared to not gesturing, and such a gestural benefit was not found for right-hand gestures. This gestural benefit of left-hand gestures was stronger for people with stronger right-hemispheric involvement for speech production in explanation tasks as inferred via the mouth asymmetry technique. We propose that hand matters for the gestures’ self-oriented functions. That is, gestures’ benefit for some linguistic tasks can be specific to one hand: left-hand gestures help speakers understand abstract concepts by mapping them onto concrete physical events in the form of metaphor, a process which particularly involves the right hemisphere.

## Supplementary Material

10.1037/xlm0000337.supp

## Figures and Tables

**Table 1 tbl1:** Parameters Estimates for the Model With the Main Effects and Interaction Between Hand Free and Presence/Absence of Spontaneous Gesture on Metaphoricity in Experiment 1

Fixed effects	Estimate	*SE*	*t* Value
(Intercept)	.891	.143	6.201
Left hand	–.291	.143	−2.039
Gesture present	.113	.133	.845
Left hand:gesture present	.395	.163	2.423
*Note.* “Right hand” and “gesture absent” were the reference categories.			

**Table 2 tbl2:** Tukey Contrasts for the Model With the Main Effects and Interaction Between Hand Free and Presence/Absence of Spontaneous Gesture on Metaphoricity for the Left Hand and the Right Hand (Experiment 1)

Contrasts	Estimate	*SE*	*z* Value	*p* Value
Left-hand gesture present vs. absent	.508	.118	4.293	<.001
Right-hand gesture present vs. absent	.113	.133	.845	.827

**Table 3 tbl3:** Parameters Estimates for the Model With the Effect of Gesturing Hand on Levels of Metaphoricity

Fixed effects	Estimate	*SE*	*t* Value
(Intercept)	1.226	.088	13.836
Left-hand gesturing	.182	.061	2.989
Right-hand gesturing	.106	.064	1.640
*Note.* “No-hand” condition was the reference category.			

**Table 4 tbl4:** Tukey Contrasts for the Model With the Effect of Gesturing Hand on Levels of Metaphoricity

Contrasts	Estimate	*SE*	*z* Value	*p* Value
No-hand–left-hand gesturing	−.182	.061	−2.989	.007
Right-hand–left-hand gesturing	−.076	.062	−1.229	.435
No-hand–right-hand gesturing	−.106	.064	−1.640	.228

## Footnotes

1 In the current study, *gestures* mainly refer to representational gestures according to the McNeill (1992) taxonomy. Representational gestures iconically depict shape, motion, and action or deictically indicate locations and directions. Speakers can also use gestures to express abstract content metaphorically (e.g., moving a palm-up open hand away from the body can express the abstract action of “conveying a message,” depicted as an object on the palm moving away from the body).

2 To illustrate how the 0–2 metaphoricity coding has been used, consider the following explanations generated for the phrase “to spill the beans”: (a) “To spill the beans is to tell someone a secret or gossip” was coded with 0 because the explanation includes the meaning of the expression only. (b) “To spill the beans means to let something out, to tell someone something perhaps that you shouldn’t been telling them. I guess the beans like information make a mess once spilling them” was coded with 1 because there is an implicit reference to the beans representing the information. (c) “To spill the beans is to tell someone something that you were not meant to tell. Something, which was confidential, private, and the beans represent the information that was private and by spilling them you are telling the news” was coded with 2 because it includes an explicit mapping between the source and target domains, and participant mentions the representation of each concept.

## A The Stimuli for the Gesture Elicitation Task

### Metaphorical Phrases for Explanation Task for Gesture Elicitation

To dodge the bullet
To fall back down to earth with a bump
To get back in the saddle
To lead someone up to the garden path
To set your sights higher
To sit on the fence
To spill the beans
To spin a yarn
To swim against the tide
To tie up loose ends

## B The Stimuli for the Metaphorical Explanation Gesture Elicitation Task and the Mouth Asymmetry Task

The items in parentheses are reserve items used when the participants did not know the main items.

### Metaphorical Phrases for Main Explanation Task for Gesture Elicitation

To burst someone’s bubble
To cross that bridge later
To dodge the bullet
To fall back down to earth with a bump
To get back in the saddle
To get hot under the collar
To hold all the cards
To leave a bad taste in the mouth
To look on the bright side
To sit on the fence
To skate on thin ice
To spill the beans
To stand your ground
To take the bull by the horns
To tie up loose ends
To turn a corner
To turn the tables
Water under the bridge

### Metaphorical Phrases for the Mouth Asymmetry Task

To pour oil onto the fire
To set your sights higher
To spin a yarn
(To hit the nail on the head)

### Concrete Phrases for the Mouth Asymmetry Task

To pour oil into the pan
To put a shelf higher
To spin a golf ball
(To hit someone on the head)
