# Supplementary material for: Hand Matters: Left-Hand Gestures Enhance Metaphor Explanation
Source: J Exp Psychol Learn Mem Cogn. 2017 Jan 12;43(6):874–86. doi: 10.1037/xlm0000337 (PMC5447392; doi:10.1037/xlm0000337)
Supplement: Supplementary file 1 [file zfv010163454so1.doc]

**Supplemental Materials**

**Hand Matters: Left-Hand Gestures Enhance Metaphor Explanation**

**by P. Argyriou et al., 2016, *JEP: Learning, Memory, and Cognition***

**http://dx.doi.org/10.1037/xlm0000337**

**Text S1**

The following inventory was adjusted from Oldfield (1971).

**The modified Edinburgh Handedness Inventory**

Please indicate your preference in the use of your hands in the following activities by circling the appropriate option. If you are indifferent, select “Either”.

| 1. With which hand do you normally write? | left | either | right |
| --- | --- | --- | --- |
| 2. With which hand do you draw? | left | either | right |
| 3. Which hand would you use to throw a ball to hit a target? | left | either | right |
| 4. With which hand do you use your toothbrush? | left | either | right |
| 5. Which hand holds a knife when you are cutting things? (not with a fork) | left | either | right |
| 6. Which hand holds the thread when you are threading a needle? | left | either | right |
| 7. When you strike a match, which hand holds the match? | left | either | right |
| 8. When you open a box, which hand holds the lid? | left | either | right |
| 9. Which hand holds the spoon when you are eating a soup? | left | either | right |
| 10. With which hand do you use scissors? | left | either | right |
| 11. Which hand is at the upper part of the broom? | left | either | right |
| 12. Which hand holds the hammer when you are driving a nail? | left | either | right |

**Text S2**

**Coding Manual for Metaphoricity Levels in the Metaphorical Explanation Task**

(Developed by Paraskevi Argyriou and Sotaro Kita)

You (the coder) will need to code the metaphoricity of the explanations that 31 participants (orally) gave for a total of 18 metaphorical idiomatic expressions, such as “to spill the beans”. The level of metaphoricity is measured based on whether the explanations include an explicit link between the literal and metaphorical meanings, that is, whether participants explicitly referred to the metaphorical mapping between the source and target domains of the conceptual metaphor underlying each idiomatic expression. According to the conceptual metaphor theory (Lakoff & Johnson, 1980a, 1980b), metaphor is a matter of conceptualizing one conceptual, abstract domain (target domain) in terms of another, concrete (source domain). For example, “time passing” is often understood in terms of “motion”, hence expressions such as “the time is here… the time for action has arrived”.

The metaphoricity of each explanation was rated on a three-point scale (0 to 2) using the following guidelines: a ‘‘0’’ rating indicated that the explanation did not contain words or phrases referring to the source domain of the relevant conceptual metaphor, therefore there was no metaphorical cross-domain mapping; a rating of ‘‘1’’ indicated that the explanation contained words or phrases that might be construed as references to the source domain, but the references were ambiguous or vague, and the mapping between the two domains implicit or underspecified; a rating of ‘‘2’’ indicated that the explanation contained words or phrases that explicitly and clearly refer to the source and target domains, and the mapping was explicit.

Exemplar coding for the expression “To dodge the bullet”

1. To dodge the bullet, means when you have a situation where you might be under some kind of danger or attack or something like that. Αnd you find a way to to save yourself from that. And it's pretty self-evident that if a bullet's coming towards you, it's gonna harm you if it touches you, or it hits you. So you you run, and jump out the way.

This explanation is coded with “0” because it is too concrete (referring only to the source domain), and the speaker does not mention how “bullet” and “dodge” represent abstract concepts, "to avoid something dangerous".

1. Τo dodge the bullet means to sort of avoid something bad that could have happened to you. Τhe bullet representing the bad thing because bullets can kill you and to dodge the bullet that means you don't get shot.

This explanation is coded with “1” because it vaguely refers to the metaphorical mapping for "bullet" (= “bullet representing the bad thing”). The representation of “dodging” is quite concrete (= “you don’t get shot” rather than “avoiding something dangerous”).

1. To dodge the bullet means to avoid consequence, that’s sort of bad consequence of one of your actions. And the bullet signifies the consequence, which is sort of punishment. And so to dodge means, you avoid it.

This explanation is coded with “2” because it explicitly refers to the two key metaphorical mappings (= “bullet signifies consequence”, “dodge means avoid”)

Specific coding issues for the application of the coding to the speech data:

1. Coding is done in Microsoft Excel file, where each raw includes the verbal response for each metaphorical explanation. You add the number 0, 1, 2 in the column named “Metaphoricity Rating”.
2. We are not coding for accuracy. In some cases participants give an “unconventional” metaphorical mapping, which is “wrong” from the viewpoint of dictionary definitions. However, this should not affect the metaphoricity coding. If the explanation includes the mapping and the representation of the concepts, even though this might be different from what you (the coder) have in mind or the dictionary definitions, you should code it with a “2”. For example, the explanation below should be coded with “2” because it explicitly represents the concepts (= “bullet is the main important thing”, “to dodge is to try to get out”) even if they are not the right ones according to the dictionary.

E.g., “Τo dodge the bullet. Well, to dodge, well the bullet is the main important thing, which is happening in the current moment, if you were trying to dodge it, it means that you're trying to get out having to think about the important issue of yeah I think, possibly.”

1. The length of the explanation is not necessarily related with how elaborate the explanation is in terms of metaphoricity; in some cases short descriptions include the representation of each concept and the metaphorical mapping, thus may be coded with a “2”. (Short descriptions may lack examples of situations for which the expressions can be used, which are not relevant for the coding)
2. The order that the representations should not affect the coding. So, for example in the explanation of the expression “to dodge the bullet” you might see the representation of the bullet first and then the one for dodging, or vice-versa.
3. If a response includes the meaning of the expression only (i.e., “to spill the beans is to tell a secret”), then it should be coded with a “0”.

**Text S3**

**R-Code for the models and comparisons reported in Experiment 1**

**Maximal model run and reported**

model.1 = lmer(Metaphoricity ~ LeftHandFree*GesturePresent + (1 + LeftHandFree*GesturePresent|Participant) + (1 + LeftHandFree*GesturePresent|Item), data = META1, REML= FALSE)

**Model with the main effects of gesture handedness and spontaneous gesture presence/absence**

model.2 = lmer(Metaphoricity ~ LeftHandFree + GesturePresent + (1 + LeftHandFree*GesturePresent|Participant) + (1 + LeftHandFree*GesturePresent|Item), data = META1, REML= FALSE)

**Model comparison**

anova(model.2, model.1)

**Tukey contrasts**

META1$handgestureinteraction<- interaction(META1_R_v2$LeftHandFree, META1v2$GesturePresent)

model.1comp = lmer(Metaphoricity ~ handgestureinteraction + (1 + LeftHandFree*GesturePresent|Participant) + (1 + LeftHandFree*GesturePresent|Item), data = META1, REML= FALSE)

summary(glht(model.1comp, linfct = mcp(handgestureinteraction= "Tukey")))

**Text S4**

**Coding manual for maximum mouth opening laterality**

(Developed by Sotaro Kita, Sarah Aldgate, Heather Golden, and Paraskevi Argyriou)

1. During speech the mouth opens and closes repeatedly without full closure except for the beginning and the end. We measure the laterality of the mouth (right, left, equal) at each maximum opening.
2. The coder selects one of the following options: the right side opens more, the left side opens more or they open equally. You make a judgment about opening based on the vertical dimension (not horizontal). The following pieces of information help determine the judgment:

(a) You can compare the maximum distance between the upper and the lower lips on the right and the left hand side. How much of the teeth you can see in each side can be helpful. But, note that you need to take into account how straight the teeth are arranged on each side.

(b) A “pull” in the upper or lower lip in a particular direction is informative. Sometimes, the muscle around the upper or the lower lip is contracted more so than the opposite side (i.e., left vs. right). When the upper lip is pulled, it looks thinner. When the bottom lip is pulled, it looks thicker. Note, however, you need to take into account the fact that some people naturally have a lip thinner/thicker on one side than the other. In some cases the wider opening is on the opposite side of the lip that is pulled. For example, the upper left lip is pulled and the right side of the mouth opens wider (This should be coded as “the right side opens more”).

(c) How the lips are joined in the two corners of the mouth can be informative.

(d) How the lips open and close before and after the maximum opening may be informative. If one side opens sooner then that side maybe the side that opens at the maximum opening. (Note that which side opens wider may change during opening. But, we code the laterality for the maximum opening only). If one side closes sooner then that side may have been opened less wide than the other.

Specific coding issues for the application of the coding to the video recordings:

1. You do not code the mouth openings in the beginning of each trial when participants repeat the phrase to be explained (e.g., “To spin a yarn means that”).
2. You do not code the mouth when it opens for non-speaking purposes, such as smiles.

**Text S5**

**R-Code for the models and comparisons reported in Experiment 2**

**Maximal model run and reported**

model.3 = lmer(Metaphoricity ~ relevel(HandFree, "None") + (1 + HandFree|Participant) + (1 + HandFree|Item), data = META2, REML= FALSE)

**Null model with no fixed effect factor**

model.3.null = lmer(Metaphoricity ~ 1 + (1 + HandFree|Participant) + (1 + HandFree|Item), data = META2, REML= FALSE)

**Model comparison**

anova(model.3.null, model.3)

**Tukey contrasts**

model3comp = lmer(Metaphoricity ~ HandFree + (1 + HandFree|Participant) + (1 + HandFree|Item), data = META2, REML= FALSE)

summary(glht(model3comp, linfct = mcp(HandFree= "Tukey")))

**Text S6**

**Analysis of the mouth asymmetry data**

We tested which side of the mouth opened wider during metaphorical and concrete explanation tasks. The left-side mouth dominance index was significantly lower than zero in the concrete (*M* = -.11, *SE* = .08, range = -.09 to 0.77), *t* (30) = -2.71, *p* = .011 but not in the metaphorical (*M* = -.24, *SE* = .09, range = -1 to .77), *t* (30) = -1.39, *p* = .176, condition. That is, in the concrete condition, the right side of the mouth opened wider than the left reflecting the important role of the left hemisphere for speaking. In the metaphorical condition, the same tendency was found numerically, but was not statistically significant.

In addition, we compared asymmetry of mouth openings during concrete and metaphorical explanations. A one-way repeated-measures ANOVA performed on the left-side mouth dominance index with linguistic task as the independent variable yielded significant effect of the task, *F* (1,30) = 6.45, *p* = .016, η_p_^2^ = .18. The left-side dominance score was significantly higher in metaphorical explanations (*M* = -.11, *SE* = .08, range = -.09 to 0.77) than in concrete explanations (*M* = -.24, *SE* = .09, range = -1 to .77). This replicates the results from Argyriou, Byfield, and Kita (2015) and indicates that the right hemisphere was particular involved during metaphorical phrase explanations, providing more evidence for the Right Hemisphere Hypothesis for Metaphor (Bottini et al., 1994). It is also line with other studies showing that mouth asymmetry measurement is sensitive to the relative involvement of the two brain hemispheres in speaking tasks (Graves & Landis, 1990). Finally, it further validates the mouth opening asymmetry as an index of the hemispheric involvement in speech production during the explanation tasks used in the current study.

References

Argyriou, P., Byfield, S., & Kita, S. (2015). Semantics is crucial for the right-hemisphere involvement in metaphor processing: Evidence from mouth asymmetry during speaking. *Laterality: Assymetries of Body, Brain and Cognition, 20*(2), 191-210.

Bottini, G., Corcoran, R., Sterzi, R., Paulesu, E., Schenone, P., Scarpa, P., . . . Frith, C. D. (1994). The role of the right-hemisphere in the interpretation of figurative aspects of language - a positron emission tomography activation study. *Brain, 117*, 1241-1253. doi:10.1093/brain/117.6.1241

Graves, R., & Landis, T. (1990). Asymmetry in mouth opening during different speech tasks. *International Journal of Psychology, 25*, 179-189.

Lakoff, G., & Johnson, M. (1980a). Conceptual metaphor in everyday language. *Journal of Philosophy, 77*(8), 453-486. doi:10.2307/2025464

Lakoff, G., & Johnson, M. (1980b). *Metaphors We Live By*. Chicago: University of Chicago Press.

Oldfield, R. C. (1971). The assessment and analysis of handedness: The Edinburgh inventory. *Neuropsychologia, 9*(1), 97-113. doi:10.1016/0028-3932(71)90067-4
